# Reflux of ^15^N-labeled uric acid after intracloacal infusion in broiler chickens fed low- or high-protein diets

**DOI:** 10.1016/j.psj.2022.101724

**Published:** 2022-01-13

**Authors:** Sonja de Vries, Joost J.G.C. van den Borne, René P. Kwakkel

**Affiliations:** Animal Nutrition Group, Wageningen University & Research, P.O. Box 338, 6700 AH Wageningen, The Netherlands

**Keywords:** avian cecum, fermentation, reverse peristalsis, urinary-Nitrogen recycling

## Abstract

Reflux of urine from the cloaca into the ceca provides chickens with a mechanism for recycling of urinary-Nitrogen (**N**) in a way analogous to urea recycling in mammals. However, it is unknown if reflux has substantial relevance in current poultry husbandry, where birds are fed ad libitum and have high protein intake. To evaluate the fate of urinary-N in ad libitum-fed broiler chickens, 15-day-old broilers were assigned to a high (21.9% CP, n = 22) or low (10.2 % CP, n = 22) protein diet. At 25 d of age, 20 broilers per dietary treatment were infused into the cloaca with a pulse dose of 107 mg [1,3-^15^N]-uric acid. N-contents and ^15^N-enrichment in digesta, blood plasma, and body tissues were measured at 5, 30, 60, 90, 150, 300, 450, 600, 1,200, or 1,800 min after administration (n = 2 /time-point /diet). Two broilers per dietary treatment were infused with saline and served as control to analyze background ^15^N-enrichment. The average total recovery (% of infused (w/w)) of ^15^N from infused uric acid in all body tissues was low (2.9 ± 0.62 %), of which the largest proportion was found in carcass tissue (2.5 ± 0.60%). ^15^N-enrichment was greatest in intestinal tissues. Even at 1,200 min, ^15^N-enrichment of ceca (0.46 ± 0.169 APE) and colon (0.13 ± 0.159 APE) digesta was considerably exceeding background enrichment. ^15^N-enrichment in excess of background enrichment in cecum and colon digesta (10-fold, *P* < 0.05), and ^15^N recovery in intestinal tissues (4-fold, *P* < 0.01) were greater in birds fed the low protein diet compared with the high protein diet, speculatively pointing out differences in the occurrence of reflux, incorporation of uric acid-N derivatives in intestinal tissues by first-pass metabolism, and a prolonged digesta retention time in protein deficient birds. In conclusion, these data confirm that uric acid-N infused in the cloaca can be refluxed and used for body N-deposition, but its contribution to whole body protein metabolism in broilers is probably limited.

## INTRODUCTION

A mechanism that is believed to facilitate efficient digestion of nitrogen in birds is reverse peristalsis of the intestine, causing digesta to flow in reverse direction; a phenomenon called reflux. Reflux from the cloaca through the colon selectively delivers fluids, including urine, to the ceca (Björnhag and Sperber, 1977; Lai and Duke, 1978; Björnhag, 1989; Duke, 1989; Vergara et al., 1989; Braun, 1999; de Vries et al., 2014), providing the bird with a mechanism for recycling of urinary-Nitrogen (**N**) in a way analogous to urea recycling in mammals (reviewed by Singer, 2003). Microbiota residing in the ceca can degrade this urinary-N and use the generated ammonia for amino acid and protein synthesis (Barnes and Impey, 1974; Karasawa et al., 1988b; Karasawa, 1989a). These microbially synthesized amino acids and proteins can potentially become available to the host (reviewed by Singer, 2003; Metges, 2000, Portune et al., 2016). In this way, birds are capable of using non-protein N as source for amino acid synthesis when dietary protein supply is limited (Fenna and Boag, 1974; Klasing, 2005), although the nutritional significance of this mechanism remains unclear (reviewed by McWhorter et al., 2009).

Several authors have made attempts to quantify the proportion of urinary-N that flows into the ceca, and estimates range from 5 to 48 %, depending on the feeding state (fed vs. fasted) and protein content of the diet (Björnhag, 1989; Karasawa and Maeda, 1995a; Son and Karasawa, 2004). Prevention of retrograde flow of urine by colostomy resulted in reduced utilization of N in White Leghorn cockerels that were fed a low protein diet where 50% of the N was provided in the form of urea, presumably due to inhibition of the degradation of urea into ammonia by cecal microbiota (Karasawa, 1989b; Karasawa and Maeda, 1994, 1995a). Colostomy did not affect N utilization when birds were fed a diet with similar N content but all in the form of protein-N (Karasawa and Maeda, 1994). In contrast, cecal ligation and cecectomy, preventing not only retrograde flow of urine but restricting any flow of digesta into the ceca, increased N retention (Karasawa et al., 1997; Son et al., 1997, 2000; Son and Karasawa, 2000, 2003; Son, 2012), likely related to inhibition of cecal fermentation and the formation of potentially harmful and neuroactive metabolites (Portune et al., 2016). Although these findings suggest that reflux of urinary-N may play an important role in nutrient utilization under certain circumstances, quantitative studies on the contribution of urinary uric acid-N, the major end-product of protein catabolism in birds, to protein metabolism in poultry are lacking. Nevertheless, the occurrence of reflux, and the flow of urinary-N into the ceca, seems typically greater when birds are fasted or when protein-deficient diets are fed (Björnhag, 1989; Karasawa and Maeda, 1994; Clench and Mathias, 1995; Godwin and Russell, 1997; Son et al., 2002). Hence, it is unknown if reflux has substantial relevance in current poultry husbandry, where birds are fed ad libitum and have high protein intake.

To evaluate the fate of urinary-N in ad libitum-fed broiler chickens receiving diets with high or low protein contents, ^15^N-labeled uric acid was infused into the cloaca and subsequently traced for 1800 min. The objectives of this study were: 1) to identify uric acid-N reflux in the gastrointestinal tract, 2) to quantify the contribution of refluxed uric acid-N to protein deposition, and 3) to obtain first insights in the temporal pattern of reflux and distribution of uric acid-N over body tissues, in broilers fed high (**high-CP**) or low (**low-CP**) protein diets. It was hypothesized that intracloacal infused uric acid will be transported to the ceca, subjected to microbial breakdown, after which uric acid-derivatives can be absorbed and incorporated in body tissues, particularly in broilers fed a low-CP diet.

## MATERIALS AND METHODS

### Experimental Design

Forty-four 15-day-old broilers were assigned to the high-CP (21.9% CP, n = 22 replicate birds) or low-CP (10.2 % CP, n = 22) diet. At 25 d of age, 20 broilers per dietary treatment were infused in the cloaca with a single pulse dose of [1,3-^15^N]-uric acid. N-contents and ^15^N-enrichment in intestinal contents, blood plasma, and body tissues were measured at 5, 30, 60, 90, 150, 300, 450, 600, 1,200 or 1,800 min after administration (n = 2 per time-point per diet). Two broilers per dietary treatment received saline instead of ^15^N-uric acid and served as control to analyze background ^15^N-enrichment.

### Birds, Housing, and Diets

The experiment was conducted at the research facilities of Wageningen University & Research, and experimental procedures were approved by the Animal Care and Use Committee (DEC) of Wageningen University & Research (2008049.b).

A total of 75 one-day-old non-vaccinated male broiler chickens (average BW: 40.3 g; Ross 308, Aviagen Group, Newbridge, United Kingdom) obtained from a commercial hatchery (Morren Kuikenbroederij B.V., Lunteren, The Netherlands) were housed in 6 floor pens (approx. 2 m2 each) with 12 (3 pens) or 13 (3 pens) birds per pen. Pens were bedded with saw dust. All birds were fed a pelleted starter diet until d 15 (Table 1).

**Table 1:** Ingredient and nutrient composition of starter and experimental broiler diets, formulated to contain high (high-CP) or low (low-CP) crude protein contents.

Item	Starter (d 1 to 15)	High-CP (d 15 to 25)	Low-CP (d 15 to 25)
Ingredient, g/kg as-fed			
Maize	287	287	445
Wheat	290	290	400
Soybean meal	330	330	30
Cellulose	-	-	40
Soybean oil	52	52	25
Diatomaceous shell powder	-	-	5.0
Limestone	15	15	15
Mineral and vitamin premix^1^	5.0	5.0	5.0
Monocalcium phosphate	13	13	16
Potassium bicarbonate	-	-	14
Salt	2.5	2.5	2.8
Sodium bicarbonate	2.0	2.0	1.8
L-Lysine HCl	1.2	1.2	-
D-Methionine	2.0	2.0	-
L-Threonine	0.4	0.4	-
Calculated nutrient composition^2^			
AME, kcal/kg^3^	2845	2845	2846
Dry matter^4^	884	884	881
Neutral detergent fiber	88.3	88.3	89.0
Crude protein^4^^,^^5^	219	219	102
Digestible lysine^6^	10.4	10.4	2.3
Digestible methionine + cysteine^6^	7.5	7.5	2.9
Calcium	8.9	8.9	8.8
Available phosphorus	4.0	4.0	4.0

1 Provided per kilogram of diet: Vitamin A (retinyl acetate), 12,000 IU; vitamin D_3_ (cholecalciferol), 2,400 IU; vitamin E (DL-α-tocopherol), 30 IU; vitamin B_2_ (riboflavin), 7.5 mg; vitamin B_6_ (pyridoxine-HCl), 3.5 mg; vitamin B_1_ (thiamin), 2.0 mg; vitamin K (menadione), 1.5 mg; vitamin B_12_ (cyanocobalamin), 20 μg; choline chloride, 460 mg; antioxidant (oxytrap PXN), 125 mg; niacin, 35 mg; pantothenic acid (D-calcium pantothenate), 10 mg; biotin, 0.2 mg; folic acid, 1 mg; Mn, 85 mg, as MnO; Fe, 80 mg, as FeSO_4_; Zn, 60 mg, as ZnSO_4_; Cu, 12 mg, as CuSO_4_; I, 0.8 mg K, as KI; Co, 0.4 mg, as CoSO_4_; Se, 0.1 mg, as Na_2_SeO_3_.

2 Calculated composition (g/kg, as-fed) based on data from CVB (2007), unless indicated otherwise.

3 Apparent metabolizable energy for broiler chickens (11.9 MJ/kg).

4 Analyzed values.

5 N × 6.25 (ISO 5983:1999).

6 Apparent total tract digestible amino acids for broiler chickens.

At d 15, the 54 most uniform chickens, based on BW and health status, were selected and housed individually in metabolism cages (0.4 × 0.3 × 0.4 m) with wired floors, to prevent coprophagy. Birds were assigned randomly to one of the 2 experimental diets, formulated to have high or low crude protein (**CP**) contents (n=27 per treatment; Table 1). After a gradual transition from starter diets to the experimental diets between d 15 and 17, birds were allowed to adapt to experimental diets for 7 d, until d 25. At d 25, 24 birds per treatment of average weight and in good health, were selected for the experiment and randomly assigned to control- (n = 2 per treatment) or ^15^N-uric acid infusion (n = 20 per treatment) groups.

Feed and water were available ad libitum during the experiment. Diets were fed as pellets and formulated to meet or exceed nutrient requirements for broiler chickens (CVB, 2007, Table 1). Diets contained fairly high fiber contents and ingredients were coarsely ground, to facilitate development of the gastrointestinal tract and (reverse) peristalsis. Ambient temperature was controlled to be 30°C at d 1 and gradually decreased to 25°C at d 18. Photoperiod was 18L:6D during the whole experiment, with lights on at 3.00 am and a minimum light intensity of 20 lux at bird level.

Feed intake was recorded per pen (d 1–14) and per bird (d 15–25) throughout the experiment. Body weight was recorded at d 1 (pen level), 15, and d 25 (individual level).

### Intracloacal Stable Isotope Administration and Sampling Procedures

At 25 d of age, a solution of [1,3-^15^N_2_]uric acid (C_5_H_4_^15^N_2_^14^N_2_O_3_, 99 atom %, Sigma Aldrich Chemie B.V., Zwijndrecht, The Netherlands) in saline (191 mg [1,3-^15^N_2_]uric acid per g) was slowly infused into the cloaca, as described by Felföldi et al. (2005). Briefly, a pipette with a disposable blunt-ended tip was used to administer 500 µL of the solution (i.e., 107 mg [1,3-^15^N_2_]uric acid). At 5, 30, 60, 90, 150, 300, 450, 600, 1,200 or 1,800 min after administration, two chickens per dietary treatment and time-point were blood-sampled (approx. 1.5 mL) from the wing vein of the left wing with a heparin-filled needle. After blood-sampling, the chickens were euthanized by injection of T61 (Intervet, Boxmeer, The Netherlands) in the right-wing vein. Blood was transferred to 2 mL Eppendorf tubes, centrifuged immediately (5 min at 10,000 rpm), and plasma was stored at −20°C. Digesta was collected per segment, from ileum (from Meckels’ Diverticulum to ileocecal junction), ceca, and colon by water-flushing (Kadim and Moughan, 1997) and stored at −20°C, for time-points 5 to 1,200 min. Livers, cleaned intestinal tissues from ileum, ceca, and colon, and the remainder of the carcasses were collected and stored at −20°C.

### Analytical Methods

Prior to chemical analyses, intestinal tissues and liver samples were freeze-dried. Intestinal tissues were defatted (ISO 6492:1999) and fat contents were recorded. Intestinal tissues and liver samples were ground by a rotor mill (Retsch ZM 100, Haan, Germany) at 18,000 rpm using a 1 mm screen, followed by a mixer mill (Retsch MM 2000, Haan, Germany) for 2 min at the maximum amplitude. The remainder of the carcasses was collected in 3 L high-model glass beakers and approximately 500 mL of doubly distilled water were added to each beaker. Carcass samples were autoclaved for 10 h at 130°C and 2 atmosphere. Each carcass was homogenized using an Ultra Turrax (T45 model, IKA Werke GmbH & Co. KG, Staufen im Breisgau, Germany) and subsamples were taken by a 5 mL syringe without needle. Subsamples were deposited in 25 mL beakers with a 4 × 4 cm polyethylene bag inside, frozen at −20°C and freeze-dried. Subsequently, samples were ground by a rotor mill (Retsch ZM 100, Haan, Germany) at 18,000 rpm using a 1 mm screen.

All chemical analyses were performed in duplicate. Diets were analyzed for contents of DM (AOAC, 2005; method 930.15) and N (Kjehldahl method (ISO 5983:2009)). Intestinal tissues, liver, and carcass samples were analyzed for contents of DM (AOAC, 2005; method 930.15). Plasma samples were analyzed for contents of uric acid using a commercial test kit (10694, Human GmbH, Wiesbaden, Germany).

Prior to N and ^15^N-enrichment analyses, plasma protein was precipitated by adding 30 µL of a sulfosalicylic acid solution (35% wt/v) to 200 µL plasma, mixing, and centrifugation (5 min at 15,000 rpm). Subsequently, precipitated plasma protein samples were freeze-dried. Nitrogen (Dumas method; AOAC, 2005; method 990.03) and ^15^N-enrichment of intestinal tissues, liver, carcass, and precipitated plasma samples were analyzed by combustion-isotope ratio mass spectrometry, using a Delta C continuous-flow isotope ratio mass spectrometer (Finnigan MAT, Bremen, Germany).

### Calculations and Statistical Analysis

Gain to feed (**G:F**) ratio for the experimental period from d 15 to 25, was calculated as BW gain divided by feed intake.

Relative organ weights were calculated as dry organ weights per 100 g BW. ^15^Nitrogen-enrichment (expressed as atom percentage excess ([**APE**) was calculated from ^15^N-enrichment in samples minus the background enrichment as measured in control birds infused with saline (average of 2 birds per treatment). Recovery of ^15^N-uric acid was calculated as the ^15^N-enrichment in excess of background enrichment × the quantity of N in samples, divided by quantity of ^15^N infused × 100%. Total body N and ^15^N were calculated as the sum of N or ^15^N in intestinal tissues, liver, and carcass.

Bird was the experimental unit for statistical analysis. Body weight, feed and N intake, organ weight and composition, and plasma uric acid concentrations were analyzed using a general linear model (PROC GLM, SAS version 9.4, SAS Institute Inc., Cary, NC), with diet as fixed effect. The effects of time-point after ^15^N-uric acid infusion and its interaction with dietary treatment were tested but found not to be significant and excluded from the model. Differences among means were tested using type III sums of squares.

Temporal patterns of ^15^N-enrichment and recovery of ^15^N-uric acid in body tissues, plasma, and digesta were first analyzed graphically, and it appeared difficult to describe the data with either linear or nonlinear models. To verify the absence of time-point × diet interactions, effects of diet were evaluated for discriminate phases (t = 0 to 200 min, t = 200 to 600 min, or t = 600 to 1,800 min). Diet effects were consistent among phases and hence, data were pooled over all time-points. Subsequently, ^15^N-enrichment in plasma and digesta were analyzed using a general linear mixed model (PROC MIXED, SAS version 9.4, SAS Institute Inc., Cary, NC), with diet as fixed effect and fitting random slopes for the various time-points estimating variances separately by diet to adjust for heteroscedasticity. Differences among means were tested using type III sums of squares. Correlation between ^15^N-enrichment in various body tissues and digesta of individual birds were expressed as Pearson's correlation coefficients (*r*; PROC CORR, SAS version 9.4, SAS Institute Inc.).

Recovery of ^15^N-uric acid in body tissues were analyzed using a generalized linear mixed model (PROC GLIMMIX, SAS version 9.4, SAS Institute Inc., with diet as fixed effect and fitting random slopes for the various time-points, assuming a beta-distributed error for the response variable and a logit link function. Degrees of freedom were approximated using the Kenward-Rogers method. Differences among means were tested using type III pseudo-likelihood ratio statistics.

Model assumptions and goodness of fit of the models were evaluated through the distribution of conditional Pearson residuals, and the ratio of the obtained generalized Chi-square to degrees of freedom. Data are presented as (back-transformed) estimated means and pooled standard error of means (**SEM**), unless indicated otherwise. Differences among means with *P* < 0.05 were accepted as representing statistically significant differences.

## RESULTS

Final BW, feed and N intake, and G:F were smaller for birds fed the low-CP diet compared with birds fed the high-CP diet (Table 2). Relative carcass weights were greater (+0.8% of BW, *P* < 0.001) and relative liver weights were smaller (−0.8% of BW, *P* < 0.001) for birds fed the low-CP diet, whereas relative intestine weights did not differ between dietary treatments (*P* = 0.756, Table 3). Nitrogen contents of carcasses were smaller (−1.7 g/100 g DM, *P* < 0.001) in birds fed the low-CP vs. high-CP diet, whereas N-contents of intestinal tissue and liver were not affected by dietary treatment. Plasma uric acid concentrations did not differ between dietary treatments (*P* = 0.383).

**Table 2:** Body weight (BW), feed and nitrogen (N) intake, and gain to feed ratio of male broiler chickens fed diets with high (21.9%; high-CP) or low (10.2%; low-CP) protein contents from 15 to 25 d of age^1^.

Item	High-CP	Low-CP	Pooled SEM	Model *P*-value^2^
n^3^	22	22		
Initial BW, g	509.7	509.2	7.03	0.966
Final BW^4^, g	1299.2	747.0	23.16	<0.001
Feed intake, g	952.7	662.7	28.16	<0.001
N intake, g	33.4	10.8	0.71	<0.001
Gain: feed ratio^4^	0.83	0.35	0.013	<0.001

1 All birds were fed the same starter diet from 1 to 14 d of age and gradually adjusted to the experimental diets between 15 and 17 d of age.

2 Model established *P*-value for the fixed effect of diet (high-CP vs. low-CP).

3 Number of replicate observations (individual birds), unless indicated otherwise.

4 One missing value for final BW and G:F, resulting in pooled SEM of 23.70 g (final BW) and 0.013 g/g (G:F) for birds fed the low-CP diet (n = 21).

**Table 3:** Carcass and organ weight and nitrogen (N) contents, and plasma uric acid concentrations, in male broiler chickens fed diets with high (21.9%; high-CP) or low (10.2%; low-CP) protein contents^1^, at 25 d of age.

Item	High-CP	Low-CP	Pooled SEM	Model *P*-value^2^
n^3^	22	22		
Total body N, g/ 100 g BW	9.2	7.4	0.10	<0.001
Carcass				
Absolute weight, g DM	373.9	255.7	9.03	<0.001
Relative weight, g/ 100 g BW	95.3	96.1	0.15	<0.001
N content, g/ 100 g DM	9.0	7.3	0.11	<0.001
Liver				
Absolute weight, g DM	11.7	5.9	0.32	<0.001
Relative weight, g/ 100 g BW	3.0	2.2	0.07	<0.001
N content, g/100 g DM	11.5	10.4	0.21	<0.001
Intestine				
Absolute weight, g DM	6.9	4.5	0.63	0.009
Relative weight, g/ 100 g BW	1.7	1.7	0.13	0.756
N content, g/ 100 g DM	13.7	13.8	0.12	0.589
Plasma uric acid^4^, µmol/L	343.8	321.6	17.85	0.383

1 Diets were fed from 17 to 25 d of age. Preceding the experimental diet, all birds received the same starter diet from 1 to 14 d of age and were gradually adjusted to the experimental diets between 15 and 17 d of age.

2 Model established *P*-value for the fixed effect of diet (high-CP vs. low-CP).

3 Number of replicate observations (individual birds), unless indicated otherwise.

4 Plasma uric acid concentrations were measured 5, 30, 60, 90, 150, 300, 450, 600, 1,200, or 1,800 min after intracloacal infusion of a pulse dose of 107 mg uric acid, in 2 birds per time-point per diet (n = 20) or in non-infused birds (n = 2 per diet).

### ^15^N-Enrichment in Digesta, Body Tissues, Blood Plasma, and Recovery of 15N From Infused Uric Acid

Although ^15^N analyses were satisfactory, seven samples were excluded from data analysis because these showed unrealistically high values, by differing more than 500% from the previous and following data point. Four of those were samples from the 5 min time-point (in carcass and intestine) in the low-CP group and one from the 30 min time-point (in carcass) in the high-CP group, which have probably been contaminated with infusate. The other sample considered a plasma sample from the 450 min time-point in the low-CP group. In addition, one of the background samples for plasma in the high-CP group was excluded because its ^15^N-enrichment deviated considerably from its replicate and did also not correspond with tissue enrichments in the same animal, suggesting an erroneous measurement.

^15^N-enrichment in livers, intestinal tissues, and digesta was greater (*P <* 0.05) and that in carcass tended to be greater (carcass, *P =* 0.059) in birds that were fed the low-CP diet vs. the high-CP diet, whereas no difference was found in plasma (data not shown). In control birds that did not receive the pulse dose of ^15^N-uric acid, background ^15^N-enrichment in body tissues, blood plasma, and digesta was numerically greater in birds fed the low-CP diet than birds fed the high-CP diet (Appendix, Table A1). In general, ^15^N-enrichment in body tissues and digesta of infused birds, exceeded background enrichment (i.e., ^15^N-enrichment in control birds), except for ileum digesta (Table 4).

**Table 4:** Recovery (% (w/w) of dosed) of ^1^^5^N in body tissues and ^1^^5^N-enrichment in excess of background enrichment (atom percentage excess, APE) in blood plasma and digesta during 1,800 min after intracloacal infusion^1^ of a pulse dose of 107 mg ^1^^5^N-labeled uric acid in male broiler chickens fed diets with high (21.9%, high-CP) or low (10.2%, low-CP) protein contents^2^ at 25 d of age.

Item	High-CP	SEM^3^	Low-CP	SEM^3^	Model *P*-value^4^
n^5^	20		20		
^1^^5^N-recovery in body tissues^6^^,^^7^	2.67	0.918	3.48	0.977	0.516
Carcass	2.50	0.888	2.84	0.928	0.779
Liver	0.03	0.013	0.02	0.013	0.782
Intestinal tissues	0.15	0.091	0.61	0.100	<0.001
Plasma ^15^N-enrichment^8^	4.7e^−4^	2.27e^−4^	1.0e^−4^	2.32e^−4^	0.265
Digesta ^15^N-enrichment^9^					
Ileum	0.00	0.018	−0.01	0.018	0.9549
Cecum	0.05	0.081	0.51	0.083	<0.001
Colon	0.03	0.082	0.32	0.087	0.022

1 Measured at 5, 30, 60, 90, 150, 300, 450, 600, 1,200, or 1,800 min after intracloacal infusion of ^15^N-labeled uric acid in 2 birds per time-point.

2 Diets were fed from 15 to 25 d of age.

3 Pooled SEM for ^15^N-recovery in body tissues, group SEM for ^15^N-enrichment in plasma and digesta.

4 Model established *P*-value for the fixed effect of diet (high-CP vs. low-CP).

5 Number of replicate observations (individual birds), unless indicated otherwise.

6 Two missing values for carcass and intestinal tissues at time-point 5 min for birds fed the low-CP diet (n = 18), one missing value for carcass at time-point 30 min for birds fed the high-CP diet (n = 19).

7 Sum of ^15^N-recovery in carcasses, liver, and intestinal tissues.

8 ^15^Nitrogen-enrichment corrected for background enrichment as measured in non-infused birds, measured at 5, 30, 60, 90, 150, 300, 450, 600, 1,200, or 1,800 min after intracloacal infusion of ^15^N-labeled uric acid in 2 birds per time-point (n = 20), except for one missing value at time-point 450 min for birds fed the low-CP diet (n = 19).

9 ^15^Nitrogen-enrichment corrected for background enrichment as measured in non-infused birds, measured at 5, 30, 60, 90, 150, 300, 450, 600, or 1,200 min after intracloacal infusion of ^15^N-labeled uric acid in 2 birds per time-point (n = 18), except for one missing values at time-points 5 and 30 min in colon for birds fed the low-CP diet (n = 16); due to limiting sample material.

**Figure 1:**
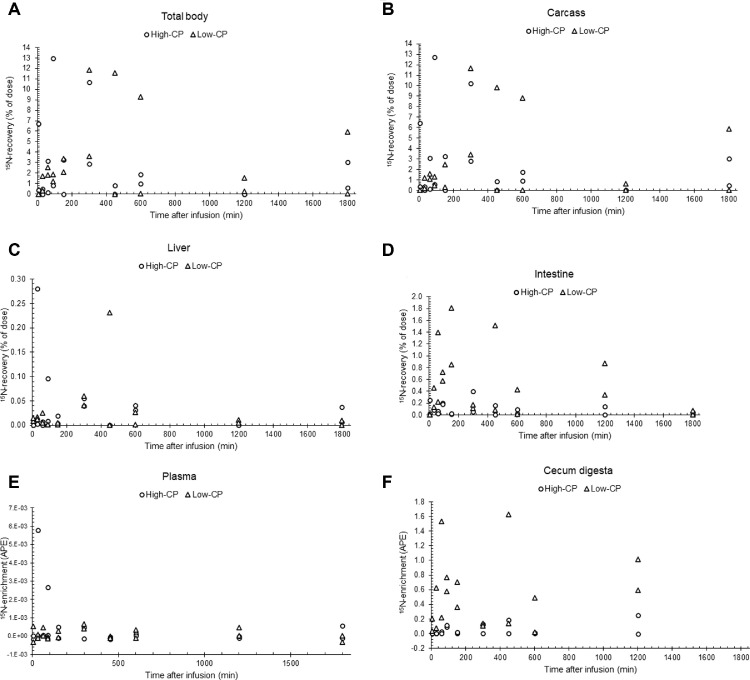
Recovery (% (w/w) of dosed) of ^15^N in body tissues (A–D) and ^15^N-enrichment (atomic percentage excess) of plasma (E) and cecum digesta (F), at 5, 30, 60, 90, 150, 300, 450, 600, 1,200 or 1,800 min after intracloacal infusion of a pulse dose of 107 mg ^15^N-labeled uric acid in male broiler chickens fed diets with high (21.9%, high-CP; circles) or low (10.2%, low-CP; triangles) protein contents at 25 d of age. Date are presented as individual observations. Diets were fed from 15 to 25 d of age.

^15^N-enrichment in excess of background enrichment (data not shown) and recovery of ^15^N from infused uric acid in intestinal tissues was greater in birds fed the low-CP diet compared with birds fed the high-CP diet, whereas no differences were found in carcasses and livers (Table 4). Temporal patterns of ^15^N-recovery between 5 and 1,800 min after infusion varied among tissues, but no clear patterns could be observed (Figure 1). Total ^15^N-recovery in body tissues (sum of ^15^N-recovery in carcass, liver, and intestinal tissues) across diets was 2.9 ± 0.62 %, with a minimum of −0.7 and maximum of 13.0 % (Table 4). ^15^N-enrichment in excess of background enrichment in plasma was generally low and highly variable, did not show notable changes over time, and did not differ between dietary treatments. ^15^N-enrichment in ileum digesta was generally low, did not exceed background enrichment in many instances, with no notable changes over time and no difference between dietary treatments. ^15^N-enrichment in cecum and colon digesta was considerably greater compared with ileum digesta. ^15^N-enrichment in excess of background enrichment in cecum and colon digesta was greater in birds fed the low-CP vs. the high-CP diet (Table 4 and Figure 1F). In individual birds, ^15^N-enrichment in excess of background enrichment in carcasses correlated well with enrichment in livers (*r* = 0.63; *P* < 0.001) and plasma (*r* = 0.56; *P* < 0.001), and to a lesser extent with colon digesta (*r* = 0.33; *P* = 0.065); but not with enrichment in intestinal tissues (*r* = 0.15; *P* = 0.388). ^15^N-enrichment in intestinal tissues correlated well with enrichment in cecum (*r* = 0.86; *P* < 0.001) and colon (*r* = 0.59; *P* < 0.001) digesta; but not with enrichment in ileum digesta (*r* = 0.11; *P* = 0.536).

## DISCUSSION

The aim of this study was to obtain some first insights in the fate of ^15^N-labeled uric acid infused in the cloaca and the temporal pattern of distribution of uric acid-N over body tissues and in digesta, in broilers fed high (**high-CP**) or low (**low-CP**) protein diets. The results indicate that indeed uric acid-N infused in the cloaca can be used for body N deposition within the 30-h measurement period, but to a limited extent.

In birds, hindgut reflux is responsible for the transport of fluid digesta, including urine, from the cloaca to the ceca; the continuous antiperistaltic contractions seem to imply that it plays a role in the digestion process (Duke, 1982). The hydration-state related control of antiperistaltic contractions in the colon suggests its main purpose is to maintain water balance of the bird (Brummermann and Braun, 1995; Braun, 1999). However, hindgut reflux is also considered a mechanism to conserve protein from the ureteral urine, which is peculiar high in birds (5 mg/L) as it is required to form a colloidal suspension with uric acid spheres and avoid coalescence (Braun, 1999). In addition, it may allow the bird to use non-protein N as source for amino acid synthesis when dietary protein supply is limited or facilitate microbial fermentation in support of energy supply (Fenna and Boag, 1974; Mortensen and Tindall, 1981; Klasing, 2005). Although the nutritional significance of this mechanism remains unclear (reviewed by McWhorter et al., 2009), the implications can be meaningful. For example, when 50% of dietary protein-N (50 g/kg diet) was replaced by urea-N, nitrogen utilization was increased by 10%-units (Karasawa and Maeda, 1994). Colostomy – preventing retrograde flow of urine – reduced N-utilization for this diet, indicating the crucial role of cecal degradation in utilization of the non-protein, urea-N, at low dietary protein supply. When 100% of N was supplied as protein (100 g CP/kg diet), however, colostomy increased N-utilization, indicating that at moderate dietary protein supply the role of ceca in N-recycling may be limited. Hence, it is unknown if hindgut reflux has substantial relevance in current poultry husbandry, where birds are fed ad libitum and have high protein intake. Moreover, the increasing trend to include more high-fiber, poorly digestible feed ingredients in animal diets – to avoid competition with (land use for) resources that can be consumed by humans directly – may increase the pertinence of N-recycling in future poultry production. Studying digesta reflux is challenging and hitherto, quantification of reflux in the hindgut and more importantly, the nutritional significance of such a phenomenon, is lacking. Although 1) the occurrence of retrograde flow of liquids from the cloaca into the ceca has been demonstrated in various birds species using exogenous tracers such as polyethylene glycol (**PEG**), Cr-EDTA, or BaSO_4_ (a.o. Björnhag and Sperber, 1977; Lai and Duke, 1978; Björnhag, 1989; Godwin and Russell, 1997), 2) the flow of uric acid from the urether into the ceca has been quantified using cecostomized chickens (Karasawa and Son, 2004), and 3) the utilization of non-protein N for body protein deposition in protein-depleted chickens has been demonstrated using urea (Karasawa and Maeda, 1994), to our knowledge no in vivo studies evaluating the fate of urinary uric acid – the major nitrogenous excretion product in birds, have been performed. By using intracloacal infusion of ^15^N-labeled uric acid we aimed to quantify the incorporation of a urinary-N-source in body tissues.

### Fate of Intracloacal Infused Uric Acid in Birds Fed Low- or High-CP Diets

Our results illustrate that after uric acid-N infused in the cloaca can be traced back in all body tissues at least up to 30 h after administration. No clear temporal pattern in ^15^N-recovery in any of the body tissues could be observed, although patterns seemed to vary among tissues. Overall, the total recovery of ^15^N from infused uric acid in all body tissues was low (2.9 ± 0.62%), of which the largest share was found in carcass tissue (mean recovery 2.5 ± 0.60%). ^15^N-enrichment was greatest in intestinal tissues, although highly variable, in accordance with the greater fractional protein synthesis rates found in gastrointestinal tissues compared with other body tissues (van der Meulen and Jansman, 1997). It should be noted that at 1,200 min, ^15^N-enrichment of digesta in colon and ceca was still considerably greater than background enrichment, suggesting that infused uric acid, or its derivatives, was still present in the intestinal lumen, either in free form or as a constituent of microbial biomass. Unfortunately, we have no data on digesta enrichment >1,200 min. Data on the retention time of digesta in the ceca of chickens are scarce, but preliminary results of our lab indicate that after feeding a pulse dose of Co-EDTA, cobalt may be found in cecal contents for >24 h (de Vries, 2018). It may thus be speculated that ^15^N-sources may be absorbed and become available to the host, even after the 30-h measurement period as monitored in this study.

The greater background ^15^N-enrichment found in birds fed the low-CP diet compared with birds fed the high-CP diet, indicates a greater natural ^15^N-enrichment in this diet, as expected from the ingredient composition with higher inclusion of wheat/maize vs. soybean meal in the low-CP diet (Harris and Paul, 1989; Wadood et al., 2019). When correcting for the initial difference in ^15^N-enrichment (^15^N-enrichment in excess of background enrichment), differences between diets were only observed for intestinal tissues, and cecum and colon digesta. The greater ^15^N-enrichment in excess of background enrichment and subsequent greater ^15^N-recovery in intestinal tissues of birds fed the low-CP diet, suggests that refluxed urinary uric acid-N derivatives may be used in first-pass metabolism by the intestinal mucosa to a larger extent in birds fed a protein-deficient diet. The greater ^15^N-enrichment in excess of background enrichment found in cecum and colon digesta may speculatively point out differences in the occurrence of reflux and a prolonged digesta retention time in protein deficient birds. This would be in accordance with the increased flow of urine into ceca in protein deficient birds, as observed previously (Björnhag, 1989).

### Using Intracloacal Infused Uric Acid to Model Urinary-N-Reflux

To our knowledge, this is the first study that investigated the fate of refluxed uric acid-N in birds. We used intracloacal infusion of ^15^N-labeled uric acid to represent urinary uric acid, the major N-containing waste product of protein catabolism in birds, and subsequently trace the uric acid derived-N in body tissues. Although the large variation in recovery of ^15^N from infused uric acid in body tissues among individual birds is in line with the large interindividual variation in retrograde flow of urine into the ceca reported previously (Björnhag, 1989), we propose that the large variation found in our study should at least be partly attributed to inaccuracies of the infusion-method. We tried to avoid immediate defecation and stimulate absorption of the uric acid infusate, by gently massaging the cloacal region during 5 min after administration, while the birds were positioned on their back. Nevertheless, excretion of the uric acid infusate cannot be excluded. Studies on the infusion-method performed in our research group, including intracloacal infusion of a contrast medium (diatrizoate) followed by qualitative verification of medium presence in the gastrointestinal tract by X-ray, indicated that only in ∼50% of the cases the pulsed-dosed iodine marker was still observed in the gastrointestinal tract 2 h after administration (de Vries et al., unpublished data). Hence, we presume that in the current study uric acid administration was unsuccessful in a proportion of the birds; resulting in large variation among birds and a potential underestimation of ^15^N-recovery. Based on the observed variation in recovery, we established cut-off values for each of the parameters (1% for recovery in carcasses and the total of body tissues, 0.2% for recovery in intestinal tissues, 0.01% for recovery in livers, 1e^−5^ APE for enrichment in plasma and 0.1 APE for enrichment in cecum and colon digesta). Out of the 40 animals that were infused in total, 7 animals did not reach the cut-off values for any of these parameters. If we assume that administration was unsuccessful in these animals and exclude them from the dataset, the mean total recovery was 3.4 ± 0.70%, with a minimum of 0 and maximum of 13%, still indicating that only a minor proportion of infused uric acid was used for body N deposition. The incidental high ^15^N-enrichment in ileum digesta, observed in the first 60 min after infusion, also seems to be an artifact of the infusion method, rather than representing ^15^N-uric acid that was actually transported from the colon through the ileum by reverse peristalsis. Nevertheless, we successfully demonstrated that intracloacal infused uric acid-N can be used for body protein deposition and the data provided useful insights in the fate of uric acid derived-N after infusion.

Although the current study, and previous literature, demonstrated that urinary (or intracloacal infused) N-sources can be incorporated in body tissues – and thus must be refluxed and subjected to conversion, the exact route and site of absorption are unclear. There is solid evidence that 1) reverse peristalsis facilitates transport of fluids, including urine, from the cloaca into the ceca (Fenna and Boag, 1974; Björnhag and Sperber, 1977; Lai and Duke, 1978; Duke, 1982; Björnhag, 1989; Braun, 1999), 2) (urinary) uric acid and urea can be degraded by cecal microbiota, leading to the production of ammonia (Mortensen and Tindall, 1981; Karasawa et al., 1988a,b; Karasawa, 1989a), 3) ammonia rapidly disappears from the ceca and appears in the cecal mesenteric vein, either directly or after conversion into amino acids and proteins by microbiota (Mortensen and Tindall, 1981; Karasawa et al., 1988b; Karasawa and Maeda, 1995b). Together, these events indicate that urinary-N may become available to the host, through degradation and conversion by microbiota. This is in line with the findings that, microbially synthesized amino acids may substantially contribute to the host's amino acid supply in several other monogastric species, especially at low dietary protein supply (Torrallardona et al., 1996,2003a,b; Newsome et al., 2011, 2020; reviewed by Metges, 2000). Although the role of cecal microbiota in the conversion of urinary-N into ammonia seems evident, the role of the cecal epithelial cells has not been excluded. For example, it has been shown that enterocytes from post-weaning piglets and sheep may use extracellular ammonia for urea synthesis (Wu, 1995; Oba et al., 2004). Also, the exact site of absorption of nutrients that become available through hindgut reflux and subsequent conversion in the ceca, has been subject to debate. Unlike other monogastric animals, significant amounts of amino acids and some sugars may be absorbed in the hindgut of chickens (Moretó and Planas, 1989; Obst and Diamond, 1989; Moretó et al., 1991; Amat et al., 1992; Planas et al., 1997; Miska et al., 2015; Miska and Fetterer, 2017). Alternatively, microbially synthesized nutrients maybe transported to the ileum by reverse peristalsis and absorbed there. Although it is generally believed that transport of contents from the hindgut to the small intestine in the fed-state is limited (Hill, 1971; Björnhag, 1989; Godwin and Russell, 1997), chromium was found in the small intestine and gizzard of fed birds after administration in the cloaca (as Cr-EDTA) (Godwin and Russell, 1997; Sacranie et al., 2012a,b). Albeit the possibility that these results originate from an artifact of the infusion method cannot be excluded, these data indicate that also the latter hypothesis could be valid.

In conclusion, the data of this study confirm that uric acid-N infused in the cloaca can be refluxed and used for body N deposition, but its contribution to whole body protein metabolism in broilers is probably limited.

## Appendix Supplementary materials

Image, application 1

## Footnotes

Supplementary material associated with this article can be found in the online version at doi:10.1016/j.psj.2022.101724.
