# Supplementary material for: Reflux of 15N-labeled uric acid after intracloacal infusion in broiler chickens fed low- or high-protein diets
Source: Poult Sci. 2022 Jan 13;101(4):101724. doi: 10.1016/j.psj.2022.101724 (PMC8866718; doi:10.1016/j.psj.2022.101724)
Supplement: Supplementary file 1 [file mmc1.docx]

**SUPPLEMENTARY MATERIAL**

Table A1. Background ^15^N-enrichment (atom percentage) as measured in non-infused male broiler chickens fed diets with high (21.9%; high-CP) or low (10.2%; low-CP) protein contents^1^ at 25 days of age^2^

| Item | High-CP | Low-CP | SD |
| --- | --- | --- | --- |
| n^3^ | 2 | 2 |  |
| Carcass | 0.3673 | 0.3677 | 0.00037 |
| Liver | 0.3670 | 0.3678 | 0.00049 |
| Intestinal tissue | 0.3668 | 0.3676 | 0.00046 |
| Plasma^4^ | 0.3675 | 0.3684 | 0.00054 |
| Ileum digesta | 0.3718 | 0.3748 | 0.00582 |
| Cecum digesta | 0.3667 | 0.3701 | 0.00298 |
| Colon digesta | 0.3672 | 0.3688 | 0.00120 |

^1^ Diets were fed from 15 to 25 d of age.

^2^ Data are presented as means and standard deviations (SD).

^3^ Number of replicate observations (individual birds), unless indicated otherwise.

^4^ One missing value for birds fed the high-CP diet (n=1).
